# Heparin-induced thrombocytopenia (HIT): Identification and treatment pathways

**DOI:** 10.21542/gcsp.2018.15

**Published:** 2018-06-30

**Authors:** Mahmoud Fathi

**Affiliations:** Aswan Heart Centre, Aswan, Egypt

## Abstract

Heparin-induced thrombocytopenia (HIT) is a major health problem, especially in cardiac surgery theaters, cardiac catheterization labs, and intensive care units. Some patients with HIT develop serious thrombotic complications like limb ischemia and gangrene, while others may not develop such complications and have only mild thrombocytopenia. Current laboratory diagnostic tools incur significant time delays before confirming HIT, therefore upon clinical suspicion, treatment of HIT should start immediately while awaiting laboratory results. This is a review of the types, phases, pathophysiology, clinical presentation and diagnosis of HIT, and its current management strategies.

## Introduction

Heparin is a proven effective anticoagulant therapy in many thrombotic conditions such as acute coronary syndrome and deep venous thrombosis, where heparin therapy is recommended after administration of thrombolytic agent to reduce mortality^1^. Combining heparin with Aspirin reduces the risk of thrombosis by 75%^2^. Although heparin is highly successful in reducing morbidity and mortality associated with thrombotic conditions, there are significant problems associated with its use. Heparin-induced thrombocytopenia (HIT) is one of the most common fatal adverse effects^3^.

HIT is a severe prothrombotic condition, occurring with fractionated and unfractionated heparin (UFH), and low-molecular weight heparins (LMWHs). It was discovered in the 1950s by Weisman and Tobin^3^. HIT carries a risk greater than 50% of developing thromboembolic events. The mortality rate is approximately 20%, and approximately 10% of patients suffer from major morbidity like amputation^4,5^.

## Types of HIT

There are two types of HIT. Type-1 is a non-immune disorder that results from the direct effect of heparin on platelet activation. Presentation starts within the first 2 days after exposure to heparin, and the platelet count normalizes without the need to discontinue heparin^6,7^.

Type-2 is a drug-induced, immune-mediated thrombocytopenia that typically occurs 4–10 days after exposure to heparin with the development of HIT II antibodies (IgG) and creates a considerably increased thrombosis risk for stroke and cardiac arrest. In general medical practice, the term ‘HIT’ refers to type-2 HIT^8–10^.

## Phases of HIT

Making a diagnosis of HIT based only on the clinical picture and low platelet count, while the results of functional assay and immunoassay are still pending, is known as “suspected HIT”. Once functional assay and immunoassay confirms the diagnosis, the patient is said to have “acute HIT”. During acute HIT the risk of thrombosis is very high. This persists up to the point at which the platelet count recovers. The period following platelet count recovery and before the washed platelet functional assay becomes negative is known as “subacute HIT A”. When the washed platelet functional assay becomes negative, but before the immunoassay becomes negative this is known as “subacute HIT B”. Finally, once anti-PF4/heparin antibodies are no longer detectable by immunoassay, the patient is said to have “remote HIT”.

**Figure 1.**
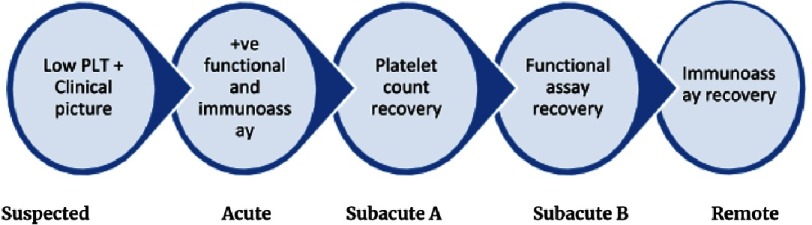
The 5 phases of HIT.

Incidence of suspected HIT is much higher than confirmed HIT, this may be explained by the high incidence of thrombocytopenia with heparin exposure in hospitalized patients and the limited specificity of clinical tests^11^. The 5 phases of HIT are shown in Figure 1.

## Incidence and risk factors

It is vital to differentiate between heparin induced thrombocytopenia (HIT) where HIT antibodies develop upon heparin exposure, and heparin induced thrombocytopenia with thrombosis (HITT) - where serious complications occur. The risk of developing HIT antibodies upon heparin exposure is about 8%, while only in 1–5% of patients exposed to heparin will develop thrombocytopenia and one third of them may suffer from arterial and/or venous thrombosis^12–14^.

The risk of developing HIT is independent of patient age, sex, heparin dose or route of administration, but differs according to the type of heparin. It is greater with bovine heparin than porcine heparin, in post-surgical (especially cardiac and orthopedic) than medical patients, and with unfractionated heparin (UFH) than low molecular weight heparin (LMWH) - but cross reactivity between both UFH and LMWH antibodies may confuse the results^4,7^. Some studies suggest that HIT occurred in 1.2% of all patients who received heparin for 4 days^15^.

## Pathophysiology

Heparin is able to activate platelets directly through interaction with platelet integrins that act as heparin receptors, especially integrin *β*III. This interaction causes increased platelet adhesion, content of *α* granules and platelet factor 4 (PF4) release, which in turn leads to a procoagulant state. PF4 binds with both circulating heparin and heparan sulphate of vascular endothelial cells forming complexes that trigger the antibody response predominantly through immunoglobulin G (IgG)^9,16–18^.

**Figure 2.**
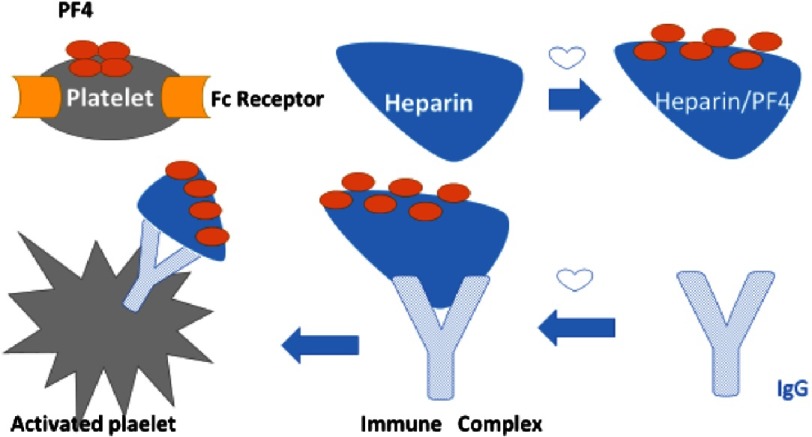
Pathophysiology of HIT.

Free IgG antibodies bind specific receptors on platelets called the Fc receptors (Fc *γ*IIA). This will lead to platelet activation. The greater the binding of Fc *γ*IIA receptors with IgG antibodies, the more intense is the platelet activation and thrombosis. The endothelium-bound IgG antibodies are able to interact with Fc *γ*IIA receptors as well, causing platelet aggregation to the endothelium and thrombogenic effect^16,19^. The pathophysiology is simplified in Figure 2.

## Clinical presentation

Clinical presentation of HIT is based on its onset after heparin exposure and the presence of complications. Unlike other causes of drug-induced thrombocytopenia, bleeding is not associated with HIT^20^. HIT type-1 is a mild transient asymptomatic thrombocytopenia that develops 2–3 days after heparin exposure and disappears quickly, patients remain asymptomatic without thrombosis^21,22^.

HIT type-2 is clinically considered when the platelet count falls by 50% or more of the baseline value and usually it develops 5–14 days of heparin exposure. It has three possible manifestations; 1) it may show HIT antibodies without thrombocytopenia (latent), 2) antibodies with thrombocytopenia but no thrombosis or 3) antibodies with thrombocytopenia and thrombosis then it is called HIT-thrombosis (HITT)^23,24^.

According to its onset HIT may be described as, a) Typical onset: occurs 5–14 days after heparin exposure, b) Rapid onset: occurs within the first 24 h of heparin exposure due to preexisting HIT antibodies from previous heparin exposure within 100 days, and c) Delayed onset: which occurs after stopping heparin treatment. It is suspected in a patient with unexplained thrombocytopenia and thrombosis 1-3 weeks after discontinuation of heparin therapy^7,25^.

## Complications

Possible complications of HIT include deep venous thrombosis (DVT), arterial thrombosis, pulmonary embolism, myocardial infarction, transient ischemic attack, stroke, skin necrosis, end-organ damage, Warfarin-induced venous limb gangrene, and death^5^.

## Differential diagnosis

(a) **Drug-induced** **thrombocytopenia**. Thrombocytopenia can be induced by drugs which can suppress the bone marrow, such as ethanol, chloramphenicol or chemotherapeutic agents, OR act via immune mechanisms like non-steroidal anti-inflammatory drugs (Aspirin, paracetamol), anticonvulsants (diazepam, valproate, phenytoin, carbamazepine), antibiotics (sulfonamides, penicillins, cephalosporins, trimethoprim), anti-diabetic agents (chlorpropamide, tolbutamide) and cardiovascular agents (thiazide diuretics, digoxin, quinidine, methyldopa)^26–29^.
(b) **Thrombocytopenia with thrombosis but with negative HIT antibodies**. This includes adenocarcinoma, pulmonary embolism, diabetic ketoacidosis, antiphospholipid antibody syndrome, thrombolytic therapy, septicemia-associated disseminated intravascular coagulation (DIC), purpura fulminans, infective endocarditis, paroxysmal nocturnal hemoglobinuria, post-transfusion purpura^26–29^.

## Diagnosis

### The pattern of thrombocytopenia

HIT-1 thrombocytopenia develops as early as 2–3 days of heparin exposure, rarely drops below 80–100 ×10^9^/L, and resolves spontaneously without stopping heparin therapy^21,22,24^. While in HIT-2, platelet count drops by 50% or more of the baseline value, with typical, rapid or delayed onset, and is usually followed by thrombotic events^23,24^. Most patients with HIT-2 have platelet count nadirs between 20 and 150 ×10^9^/L (mean nadir, 55 ×10^9^/L)^30^.

### Probability of HIT

As mentioned before, most heparin treated patients do not develop HIT, so it must be accurately predicted. The 4Ts is a clinical scoring system used to predict HIT in thrombocytopenic patients treated with heparin while waiting for laboratory results for antibodies. It assesses the extent of thrombocytopenia, timing of platelet count fall, presence of thrombosis or other complications and other causes of thrombocytopenia. A score of 0–2 is given for each item with maximum score of 8. The 4Ts score is illustrated in Table 1^30–32^.

**Table 1:** 4Ts scoring system^38^.

	Score		
	0	1	2
Thrombocytopenia: Percentage of platelet count	<30% fall OR Nadir <10 × 10^9^/L.	30–50%% fall or Nadir 10-19 × 10^9^/L.	>50% fall or Nadir ≥20 × 10^9^/L.
Time / onset of thrombocytopenia	<Day4 with no recent heparin exposure.	>Day 10 OR <Day 1 with heparin exposure between 31–100 days OR Unknown onset.	>Day 5–10 OR Day 1 with heparin exposure <30 days.
Thrombosis / complications	No	Progressive or recurrent thrombosis OR suspected thrombosis OR skin erythema.	Proven new thrombus OR skin necrosis OR acute systemic reaction after UFH bolus.
Other causes of Thrombocytopenia	Proven	Possible	Not proven

Interpreting the 4Ts scoring system with score of 0–3 means low probability of HIT antibodies (<5%), score of 6–8 means high probability (>80%), while score of 4–5 means intermediate probability (see Figure 2)^30,32^. The intermediate score of 4–5 may be caused by other conditions like sepsis, so the laboratory testing for antibodies is particularly conclusive in these patients^31^.

**Figure 3.**
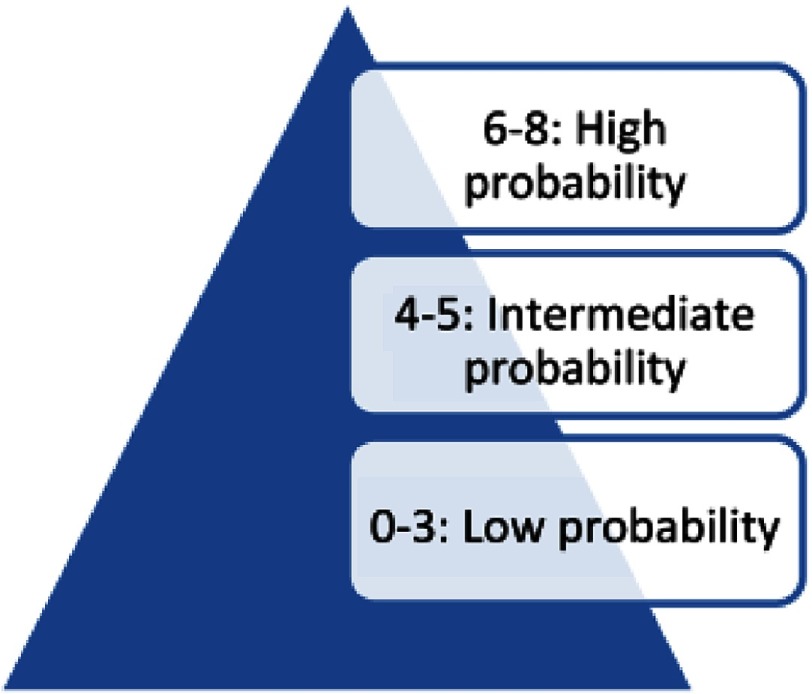
Total scores and corresponding probability of HIT.

**Figure 4.**
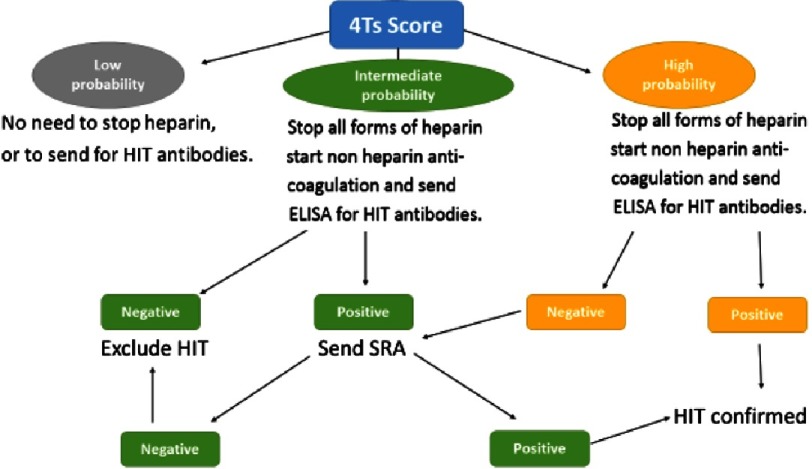
The 4Ts scoring system and subsequent actions.

A low probability 4Ts score has a negative predictive value of 99.8 % (95 % confidence interval [CI] 97.0–100.0) while an intermediate and high probability 4Ts score have a positive predictive value of 14 % (9–22) and 64 % (40–82), respectively^33^. The presence of incorrect or missing information may lead to a false 4Ts score and incorrect management decisions e.g., if platelet counts are not available, it may lead to higher 4Ts score. Some reports suggest that the 4Ts score does not perform as well in the intensive care and post-cardiac surgery settings^34–36^. Other scoring systems show promise but have not been adequately validated for clinical use^33,37^. The 4Ts scoring system is simplified in Table 1 and Figures 3 and 4.

## Laboratory confirmation

The result of laboratory confirmation of HIT usually lags behind clinical diagnosis, besides there is no assay that has complete 100% sensitivity or specificity. So laboratory assays should serve as complementary tests in the diagnosis of HIT and initial management should be based on the probability of HIT. They shouldn’t be done to routinely test for HIT antibodies in the absence of clinical features of HIT, such as thrombocytopenia, thrombosis, or heparin-induced skin lesions. Acute serum or plasma should be used in the diagnostic assays^26,39,40^.

Two types of assays are available:

***Functional assay****:* ***platelet activation or a serotonin-release assay (SRA)***

Platelet-activation assays include platelet aggregation assay and the SRA. The platelet aggregation test is rapid (2–3 h), simple and inexpensive. Its major drawback is the low sensitivity compared to SRA (30% to 50%)^39^. The SRA is the gold standard for laboratory confirmation of HIT. Its main principle is to use serotonin to radiolabel the normal donor platelets then wash them; making them very sensitive to activation by HIT serum. The patient’s serum, as well as therapeutic (0.1 U/mL) and high (100 U/mL) concentrations of heparin, are added. A result is considered positive if significant activation occurs at therapeutic levels and does not occur at high levels. SRA has high sensitivity (90% to 98%) and specificity (above 95% in early phases and 80% to 97% for late-phase platelet declines). Unfortunately, it is technically demanding, time consuming, and not readily available at most institutions^26,41^.

***Antigenic (immune) assay: enzyme-linked immunosorbent assay (ELISA)***

Complexes of PF4 with either heparin or polyvinyl sulfonate are coated on a microtiter plate, and then the patient’s serum is added. If antibodies against the PF4 complex are present, they bind together. This can be confirmed by adding a second antibody. A negative result strongly rules out the diagnosis of HIT, while a positive result needs to be confirmed by functional assay. The test sensitivity is more than 90% and specificity at 95% in early platelet decline and at 50% to 93% in late platelet decline^26^.

A potential drawback is that the antigen assays may detect the presence of clinically insignificant HIT antibodies (does not activate platelets), this concludes that clinical HIT does not always develop in patients with positive test results and that a positive ELISA with no confirmatory functional test does not support the diagnosis of HIT alone. A 4Ts score consistent with clinical presentation is needed. The ELISA test can be improved by increased specificity to detect IgG antibodies to the heparin–PF4 complex rather than detecting a combination of IgG/IgA/IgM^26,41,42^.

## Management

If the 4Ts score shows high or intermediate probability of HIT, all forms of heparin should be immediately discontinued, heparin-coated catheters should be immediately removed, and a non-heparin anticoagulant should be started to prevent thrombosis^43^.

Studies showed that starting prophylactic anticoagulants with intermediate probability HIT did not prevent thrombosis^44^, while a more recent study used prophylactic anticoagulation in patients with intermediate probability HIT and showed negative compression ultrasonography and immunoassay result^36^.

### Non heparin anticoagulation

This includes two groups of drugs direct thrombin inhibitors and indirect factor Xa inhibitors.

#### Direct thrombin inhibitors

##### Argatroban.

Argatroban is a synthetic direct thrombin inhibitor. It acts on both free and clot-bound forms of thrombin. It is metabolized in the liver with a half-life of about 40–50 min^45^.

Argatroban is administered via continuous intravenous infusion at a dose of 2 µg/kg/min. Dose adjustment is required in case of liver dysfunction (bilirubin >1.5 mg/dl) to 0.5–1.2 µg/kg/min, heart failure, anasarca and post-cardiac surgery to 0.5–1.2 µg/kg/min.

Treatment is monitored with the activated partial thromboplastin time (APTT) with target 1.5–3 times baseline. Less commonly, the dilute thrombin time or ecarin clotting time are is used for monitoring^46^.

Argatroban-treated subjects were compared to historical untreated controls in single-arm open-label studies^47^. These studies concluded that argatroban reduces the risk of new thrombus formation by 70 % compared with controls with bleeding risk of 0.99 % /day^48^.

Despite being approved for treatment of HIT, argatroban has many limitations such as being expensive, administered via continuous intravenous infusion with frequent laboratory monitoring, and dose adjustment. Also it has a narrow therapeutic index, carries a daily risk of major hemorrhage of about 1%, and does not reduce frequency of limb amputation or death^48–51^.

Being monitored by APTT, argatroban under-dosing may occur due to HIT-associated consumptive coagulopathy^52^. Argatroban also raises the international normalized ratio (INR), complicating transition to vitamin K antagonists^53^.

##### Bivalirudin.

Bivalirudin is a synthetic direct thrombin inhibitor. It has a short half-life of about 25 min. Its metabolism is less reliant on liver or kidney function than other non-heparin anticoagulants. It is administered via continuous intravenous infusion at a dose of 0.15 mg/kg/h. In cases of renal or liver dysfunction dose reduction may be appropriate. In the operating room and catheterization suite, bivalirudin is monitored by activated clotting time. Elsewhere, the APTT is used with target 1.5–2.5 times baseline. Assessment of bivalirudin in hospitalised patients was limited to retrospective studies^54,55^.

Based on large clinical trials bivalirudin has been approved for use in percutaneous coronary intervention (PCI) whether the patient has HIT or not^56^, and in cardiac surgery with or without cardiopulmonary bypass^57,58^.

##### Desirudin.

Desirudin is a recombinant hirudin. It is cleared through the kidneys with a half-life of about 2 h. It is administered via subcutaneous injection with initial dosage of 15 to 30 mg every 12 h. Desirudin is approved for thromboprophylaxis after hip arthroplasty, but it is not yet approved for treatment of HIT^59^.

#### Indirect factor Xa inhibitors

##### Danaparoid.

Danaparoid is a complex of glycosaminoglycans that have antithrombin-dependent anti-Xa activity. It is cleared through the kidneys with a half-life of 24 h. Danaparoid has a relatively more complex administration, given intravenously with an initial bolus dose calculated according to the patient’s weight <60 kg → 1500 units 60–75 kg → 2250 units 75–90 kg → 3000 units >90 kg → 3750 units, followed by infusion at 400 units/h for 4 h, then 300 units/h for 4 h, and then maintenance infusion at 200 units/h, which should be reduced in renal dysfunction to 150 units/h. Monitoring is adjusted to danaparoid-specific anti-Xa activity of 0.5–0.8 units/ml. Prophylactic-dose danaparoid should be avoided in patients with HIT because of a relatively high rate of breakthrough thrombosis^51,59^.

Danaparoid is approved for the treatment of HIT in many countries, but not in the US. In an open-label trial, patients with HIT and thrombosis were randomized to danaparoid or dextran-70. Recovery from thrombosis was greater in the danaparoid arm (56 % vs 14 %, *p* = 0.02). Despite being approved for treatment of HIT, danaproid has many limitations similar to argatroban^60^.

##### Fondaparinux.

Fondaparinux, a synthetic antithrombin-dependent factor Xa inhibitor. It is cleared through the kidneys with half-life of 17-24 h, and administered by subcutaneous (SC) injection. Dose is calculated according to body weight <50 kg → 5 mg, 50–100 kg → 7.5 mg, >100 kg → 10 mg given SC once daily. It is contraindicated in renal dysfunction and does not require routine monitoring^59^.

Despite being not approved for treatment of HIT, fondaparinux is being widely used because it does not require routine monitoring or dose adjustment, is administered subcutaneously, and has a no effect on the INR, thereby facilitating transition to outpatient therapy. Studies suggest that fondaparinux has similar efficacy and safety compared to agents approved in treatment of HIT^61–64^.

Choice of a non-heparin anticoagulant should be based on the patient’s hepatic and renal function, clinical condition, and drug availability.

For hemodynamically-stable patients with normal renal functions, fondaparinux would be the drug of choice. If the patient has renal impairment, argatropan, bivalirudin or danaparoid can be used. Argatroban is contraindicated in hepatic impairment.

For hemodynamically-unstable patients with normal hepatic functions, argatropan, bivalirudin or danaparoid are the drugs of choice. Argatroban is contraindicated in hepatic impairment^59^.

The non-heparin anticoagulation therapy should continue for at least three months in cases of HIT complicated by thrombosis. In case of isolated HIT, anticoagulation should be continued for 4-6 weeks, or until platelet recovery. Platelet recovery means a platelet count ≥150 × 10^9^/l or a rise in platelet count into a stable plateau that increases by 10 % over 3 consecutive days^46,65^.

## Transition to oral anticoagulation

Vitamin K antagonists (VKA’s), such as warfarin, should be avoided in patients with HIT until platelet count recovery because they cause depletion of protein C, which will may lead to venous limb gangrene. If a patient is on a VKA at the time HIT is diagnosed, it should be discontinued immediately and reversed with vitamin K^46,66,67^.

After platelet recovery, VKA should be initiated without loading doses and overlapped with a parenteral anticoagulant for at least five days until the INR has reaches the intended target^46^.

Shifting from argatroban to a VKA is challenging because of the effect of argatroban on the INR, as it may lead to under-anticoagulation. The overlap should be at least five days with daily INR measurement, then argatroban may be stopped when the INR exceeds 4. The INR should be re-measured 4–6 h after cessation of argatroban. If the INR is below the target range, argatroban should be resumed and the procedure should be repeated daily until the target INR is achieved on VKA alone^68,69^.

Direct oral anticoagulants (DOACs) may be used instead of VKAs after platelet count recovery. They do not require overlap with parenteral anticoagulation or routine laboratory monitoring and have a lower bleeding risk^65^.

Platelet transfusion is rarely indicated in HIT. It should be considered only in cases of life-threatening bleeding, prior to a procedure with high bleeding risk like cardiovascular procedures, or in patients with severe thrombocytopenia (<20 × 10^9^/l)^65^.

New lines of therapy include PF4 antagonists and non-pathogenic anti-PF4/heparin monoclonal antibodies which interfere with formation of PF4/heparin complexes, Fc *γ*RIIA blockers which prevent binding of HIT immune complexes to receptors on platelets, and inhibitors of splenic tyrosine kinase and Ca^2 +^ diacylglycerol-regulated guanine nucleotide exchange factor I which disrupt intracellular pathways triggered by immune complex binding^70–72^.

## HIT and cardiovascular interventions

Cardiovascular surgery is contraindicated in acute HIT and subacute HIT A. It should be postponed ideally until remote HIT or at least subacute HIT B^65^. If surgery cannot be delayed, a non-heparin anticoagulant such as bivalirudin should be used^54,58^.

Re-exposure to heparin in patients with history of HIT should be strictly intraoperative. If pre- or post-operative anticoagulation is indicated, a non-heparin anticoagulant should be prescribed^65^.

The safety of intraoperative heparin was studied in patients with subacute HIT B and remote HIT undergoing urgent cardiac surgery, none of them developed recurrent HIT^73,74^.

Preoperative plasmapheresis may be performed to remove HIT antibodies and enable intraoperative heparin^75^.

Bivalirudin is preferred for percutaneous vascular procedures in all patients with a history of HIT^56^. If bivalirudin is not available, heparin can be used in patients with subacute HIT B and remote HIT during the procedure only^65^.

HIT antibodies are present in about 10% of patients on chronic hemodialysis, but the development of HIT is uncommon in these patients (<1 %)^76,77^. In patients with a history of HIT, treatment with heparin during hemodialysis is contraindicated. Use of danaparoid, argatroban beside dialysis circuit flush with saline, regional citrate and VKA have been reported, but not compared or systematically investigated^65^.

## Conclusion

Upon exposure to heparin, many patients develop HIT antibodies. A small number of them (1–5%) develop thrombocytopenia, and even fewer develop thrombosis. The 4Ts scoring system is used clinically to predict HIT and guides immediate further management. When there is a high probability of HIT, all forms of heparin should be stopped and treatment with a non-heparin anticoagulant should be started.
